# Hidden time-patterns in cyclic human movements: a matter of temporal Fibonacci sequence generation and harmonization

**DOI:** 10.3389/fnhum.2025.1525403

**Published:** 2025-04-08

**Authors:** Cristiano Maria Verrelli, Lucio Caprioli, Marco Iosa

**Affiliations:** ^1^Department of Electronic Engineering, University of Rome Tor Vergata, Rome, Italy; ^2^Sport Engineering Lab - Department of Industrial Engineering, University of Rome Tor Vergata, Rome, Italy; ^3^Department of Psychology, Sapienza University of Rome, Rome, Italy; ^4^Smart Lab, IRCCS Santa Lucia Foundation, Rome, Italy

**Keywords:** generalized Fibonacci sequence, symmetry, automatized movement, self-similarity, golden ratio, neuroscience, physiological systems

## Abstract

Fibonacci sequences are sequences of numbers whose first two elements are 0, 1, and such that, starting from the third number, every element of the sequence is the sum of the previous two. They are of finite length when the number of elements of the sequence is finite. Furthermore, Fibonacci sequences are named generalized Fibonacci sequences when they are generated by two positive integers—called seeds—that do not necessarily equal 0 and 1. This relaxation provides the analyst with larger degrees of freedom if the elements of the Fibonacci sequences have to refer to the durations of the sub-phases of a physical movement or gesture that differ from 0 and 1. Indeed, by taking inspiration from their use of symmetric walking—where the stance duration is the sum of the double support and swing durations and, in turn, the duration of the entire gait cycle is the sum of the stance and swing durations—, generalized Fibonacci sequences of finite length have been very recently adopted to extend the resulting original walking gait characterization to gestures in elite swimmers and tennis players, by accordingly associating the durations of the sub-phases of the gesture to the elements of such sequences. This holds true within movement-automatization-allowable scenarios, namely, within scenarios in which no external disturbances or additional constraints affect the natural repeatability of movements: at a comfortable speed in walking, at a medium pace in swimming, and under no need for lateral/frontal movements of the entire body in tennis forehand execution or no wind in the serve shot. Now, in such sequences of sub-phase durations of a physical movement or gesture, the golden ratio has been further found to characterize hidden self-similar patterns, namely, patterns in which all the ratios between two consecutive elements of the sequence are surprisingly equal, thus representing a harmonic and mostly aesthetical gesture that admits a perfectly self-similar sub-phase partition in terms of time durations. In such a case, the larger scale structure within the gesture resembles the smaller scale structure so that the brain can aesthetically resort to the minimum amount of information for the movement temporal design. In the framework of how cognitive factors such as working memory and executive control facilitate motor learning and adaptation, this paper addresses, for the first time in the literature, the open problem of providing a complete mathematical understanding of the automatic generation process at the root of such hidden Fibonacci sequence-based and self-similar patterns appearing in the aforementioned cyclic human movements. Data referring to walking and tennis playing are used to illustrate the effectiveness of the proposed approach.

## 1 Introduction

Motor learning is expressed by a relatively permanent change in movement performance, resulting from training or previous experience in the situation. Indeed, cognitive processes, such as perception, attention, reasoning, memory, and problem-solving, operate to help produce skilled movement performance and are all involved in skill learning (Magill and Anderson, 2010). Automaticity of movements occurs when subjects no longer need to pay attention to the act itself during the movement execution. The natural and non-conscious coordination of a set of body segments is thus allowed to form a smooth and efficient execution (Kibler and Sciascia, 2004). In particular, in sports, to learn specific skills and effectively execute gestures, coordination is fundamental and is developed through training (Haubenstricker and Seefeldt, 1986). The number of repetitions of the gesture to be learned represents one of the necessary elements for storing information about the initial conditions, sensory feedback, and the results obtained to form and reinforce the action pattern (Schmidt et al., 2018). The performance is refined each time with each new execution; in fact, the technique is an element that can be modified and refined continuously until a relatively stable pattern is formed by which the movement can approach the desired technical model. The effectiveness of practice, defined as the number of repetitions, has been accordingly recognized as the basic elements of learning and perfecting gestures (Jonides, 2004; Lee and Genovese, 1988): learning takes place as a process dependent on subjective experience with each experience being able to significantly influence neuronal connections and brain structures, a phenomenon known as neural plasticity (Galván, 2010). Some mental abilities also can intensify and enhance perceptions so that learning can be viewed as an active process of adaptation through the acquisition of automatic stable behaviors due to both external and internal stimuli. Currently, some human movements, such as walking, are characterized by cyclic and reliable patterns. Recent studies have observed that the repeated structure is not only evident between cycles but also within each cycle, where a specific proportion is preserved. This proportion creates a larger scale structure that resembles the smaller scale structure through the generation of a self-referential loop. Indeed, this self-similarity in complex movements is mathematically associated with the golden ratio, an irrational number related to fractals and the Fibonacci sequence. In particular, the golden ratio $\phi =\left(1+\sqrt{5}\right)/2≊1.618$ is the (positive) solution to the equation *x*^2^ = 1 + *x* (Marino et al., 2020). It is related to Euclid's problem of cutting self-proportionally a given segment (namely, as the whole segment is to the greater subsegment, so is the greater to the lesser) and possesses geometric and aesthetic properties (namely, the highly aesthetical *golden rectangle* with long side *a* + *b* and short side *a* can be divided into two pieces: a similar golden rectangle with long side *a* and short side *b* and a square with sides of length *a*), making it an object of interest in computer science, art, architecture, and design. Among the mathematical properties of the golden ratio, ϕ^−1^ = ϕ−1 seems to be the most effective, as ϕ^−1^ is the limit value of the ratios between two consecutive numbers of the generalized Fibonacci sequence (Bormashenko, 2022; Rostami, 2006; Mohanta et al., 2023). Another point of view can be provided as well. The considered human movements are executed through the coordination of multiple muscles, involving simultaneous control of several degrees of freedom. The abundance of degrees of freedom allows humans to achieve the same goal through various possible patterns of muscle activations. The degrees of freedom, motor equivalence, or Bernstein's problem (Bernstein, 1967) consists of explaining how the central nervous system (particularly the brain) rapidly selects the optimal set of activation patterns. One proposed optimization strategy involves the use of fractals to account for the complexity of biological systems and motor control (Goldberger, 1996). In this case, the optimal solution is the replication of a specific schema at different levels; in fact, a fractal is a characteristic or phenomenon composed of subunits that resemble the larger scale structure of the whole unit in a recursive or self-similar manner (Mandelbrot, 1977). With this respect, the simplest example of self-similarity is the golden ratio, which is, as aforementioned, related to the Fibonacci sequence and has been measured in motor patterns (Iosa et al., 2018). In particular, very recent research efforts (Verrelli et al., 2021a), starting back from Iosa et al. (2013), have been dedicated to theoretically characterizing and explaining the experimental occurrence of golden ratio-based time-harmonic motor patterns in human walking. They involve the following: (i) the foot-off event, which typically happens at 60% to 62% of the physiological gait when a subject is walking—symmetrically and recursively—at his/her comfortable speed [approximately 4 km/h (Cavagna and Margaria, 1966)]; (ii) the ratio between the swing and the double support phase durations, which is close to the golden ratio, with this holding true even for the ratio between the stance and the swing phase durations. From a mathematical point of view, the resulting discoveries explain the existence of patterns that are implicitly defined by the golden ratio when it occurs as the ratio of gait sub-phases durations composing a generalized Fibonacci sequence. According to a similar principle, they appear not only in walking but also in running and swimming (Verrelli et al., 2021b,c, 2023) at middle-long pace. It is true that swimming does not seem to belong to CPG-based instinctive patterns, but it naturally owns a rhythmicity similar to walking and running as soon as it is induced by repetitive training for a long enough time. A high level of technique automatizes the learning of complex movements, so high/top-level athletes can avoid redundant time- and energy-consuming movements. Nevertheless, the results of the latest (Verrelli et al., 2024) confirm the existence of the same harmonic structures even for the forehand of advanced-level tennis players in comfortable conditions (i.e., with no need for lateral/frontal movements of the entire body), so they seem to allow the brain/body to optimize energy usage in temporally designing the shot. Even though tennis is not traditionally considered as cyclic as running and swimming, which are suitable examples of rhythmic activities, however, some aspects of tennis movements certainly become highly stereotyped and efficient with practice. Currently, even in the presence of unquestionable developments, the process at the root of such experimental and theoretical evidence is still far from being totally understood. Within the framework of cognitive factors such as working memory and executive control facilitating motor learning and adaptation, this paper addresses, for the first time in the literature, the open problem of:

- (i) providing an algorithmic understanding of the automatic process that generates mechanized and self-similar patterns appearing in cyclic human movements within movement-automatization-allowable scenarios, while
- (ii) simultaneously explaining the existence of mechanized cyclic movements that do not perfectly satisfy the self-similar internal partition yet.

In particular, mechanized cyclic movements that do not perfectly satisfy the self-similar internal partition happen in swimming and tennis—more often than walking, for which a large number of repetitions have been generally performed—where the disjunction between temporal symmetry and self-similarity & golden ratio occurrence as a fixed point is made more evident by the role played by the technical abilities of players in conjunction with the training level. In other words, the key idea here hinges on an original conceptual disjunction—within movement-automatization-allowable scenarios under no external disturbances or additional constraints—between (i) the *Fibonacci sequence generation* through the process of temporal symmetrization and ii) the *time harmonization* through the self-similarization step. While the former achieves ever higher levels of coordinative mechanization—based on an ever longer Fibonacci sequence—with the self-similarity playing a just asymptotic role for such a first process, the latter perfects, enhances, and refines the former while achieving minimization of the Shannon entropy (see Serrao et al., 2017 for an experimental interpretation in terms of energy-expenditure-minimization and Verrelli et al., 2021a for dynamics-on-graph interpretations) and flowering of aesthetic (highly technical) movement characteristics. The two aforementioned processes are conceived to involve an ever lower amount of information for the movement temporal design, by building ever better automatic circuits of mental dependence on memory as soon as the length of the Fibonacci sequence increases and self-similarity is additionally achieved (Section 2 and Appendix). The consequent internal evolutionary process uses recursive limits as fundamental canons of perfection while memorization induces reflective loops. Data referring to walking (Section 3) and tennis-playing (Sections 4, 5) are used to illustrate the effectiveness of the proposed approach. Implications of these findings for rehabilitation and sports training are also discussed (Section 6).

## 2 Automatic generation process

The original idea of the paper is presented in this section.

### 2.1 Generation process (steps 3–4)

With the aim of improving the readability of the section, the main logical considerations are applied to a specific step of the procedure (namely, the step corresponding to a Fibonacci sequence of length 4), before extending it to the generic step of the same procedure. To the same purpose, the description—though being back-extendable (see Remark 1)—starts directly from the step corresponding to a Fibonacci sequence of length 3. The resulting step is named steps 3–4.

#### 2.1.1 Fibonacci sequence generation through a symmetrization process (steps 3–4)

A Fibonacci sequence is one of the simplest ways of generating a sequence on the basis of a self-referential loop. For the sake of simplicity, the reader can think of the event below as the human walking gait cycle, partitioned into the double support, left swing, and right swing phases. Consider an event F consisting of three consecutive disjoint phases FA, FB, FC with durations ${\text{d}}_{FA}$, ${\text{d}}_{FB}$, ${\text{d}}_{FC}$. Apparently the duration ${\text{d}}_{F}$ of the event F satisfies


(1) $$\begin{array}{l} {\text{d}}_{F}={\text{d}}_{FA}+{\text{d}}_{FB}+{\text{d}}_{FC}. \end{array}$$

Without loss of generality, let FA be the phase with the minimum duration, namely


(2) $$\begin{array}{l} {\text{d}}_{FA}=\min\left\{{\text{d}}_{FA},{\text{d}}_{FB},{\text{d}}_{FC}\right\}. \end{array}$$

It is the double support phase (whose duration is generally smaller than the swing phase) in the recursive and consistent walking gait cycle of a healthy subject (Verrelli et al., 2021a). Then, consider the generalized (non-decreasing) Fibonacci sequence (Horadam, 1961) of length 3 associated with the aforementioned partition:


(3) $$\begin{array}{l} {\text{Fib}}_{3}:{\text{d}}_{FC},{\text{d}}_{FA}+{\text{d}}_{FB},{\text{d}}_{F}. \end{array}$$

Notice how this sequence is the one that comes from the automatic procedure of this subsection once it is directly applied to event F. Furthermore, the aggregate phase whose duration constitutes the second element of Equation 3 resembles the stance phase in human walking. Currently, the question is as follows: under which conditions, the length of the above Fibonacci sequence (Equation 3) can be increased by splitting, into the two phases FA,FB, the aggregate phase FA∪FB that appears, with its duration, as a second element of Equation 3? With this in mind and looking for a formal development, introduce the following definition. As we shall see, such a definition resembles the case of a symmetrical human walking gait cycle with left and right swing phases of equal duration.

**Definition 1**: Event F, consisting of three consecutive disjoint phases FA, FB, FC whose durations satisfy Equations 1, 2 and compose the Fibonacci sequence Fib_3_ of Equation 3, is *once-Fibonacci-left-extendable* if it is symmetrically partitioned, i.e., if the second and the first elements of Equation 3 satisfy the temporal constraint


(4) $$\begin{array}{l} {\text{d}}_{FB}={\text{d}}_{FC}. \end{array}$$

In other words, a symmetrically partitioned event F consists of a shorter phase FA and two longer phases FB, FC of equal duration. This is the key idea at the root of the argument transposition extending the self-similar (harmonic) analysis of walking to swimming and tennis playing. The resulting pattern, call it $P_{A}$, which symmetry is at the root of, will constitute the special pattern that the automatic generation process of this subsection will aim at replicating, while the length of the Fibonacci sequence is repeatedly increased. This is shown in detail by the following theorem.

**Theorem 1**: The generalized (non-decreasing) Fibonacci sequence of length 4 (extending Fib_3_ of Equation 3 to the left)


(5) $$\begin{array}{l} {\text{Fib}}_{4}:{\text{d}}_{FA},{\text{d}}_{FC},{\text{d}}_{FA}+{\text{d}}_{FC},{\text{d}}_{F}. \end{array}$$

is associated with the once-Fibonacci-left-extendable event F of Definition 1.

Proof. Just recognize that ${\text{d}}_{FC}+{\text{d}}_{FA}+{\text{d}}_{FC}={\text{d}}_{FC}+{\text{d}}_{FA}+{\text{d}}_{FB}={\text{d}}_{F}$.

To understand well the relevance of Theorem 1, we formulate the following question. Why a longer Fibonacci sequence (e.g., Equation 5 in place of Equation 3) might be associated with a higher level of coordinative mechanization in the generation of the event F? Apparently, the extension of Equation 3 to the left removes the aggregate phase FA∪FB in Equation 3 from the set of seeds and makes the elementary phase FA, in Equation 5, enter it. This means that the generation of the self-referential loops involves an increased-by-one number of elementary phases in place of an aggregate one, once the length of the Fibonacci sequence is increased-by-one. This also means that the generation of the self-referential loops will turn out to involve an ever larger number of elementary phases in place of aggregate ones, once the length of the Fibonacci sequence is repeatedly increased. In this light, coordinative mechanization is viewed as an increase in the number of physical phases of the movement that enter the set of seeds within the self-referential loop so as to reduce the number of durations to be independently determined and memorized in the design of the event. This happens at the price of an increased number of repetitions performed by the human, in which the temporal symmetrization step of Definition 1 and Theorem 1 is successfully performed. Nevertheless, there exists a connection between a sufficiently long Fibonacci sequence (i.e., a higher level of coordinative mechanization) and the golden ratio. If sequence (Equation 5) is represented through the discrete time, second-order autoregressive scalar system: *y*(*k* + 2) = *y*(*k* + 1) + *y*(*k*) (*k* = 0, 1), with $y\left(0\right)={\text{d}}_{FA}≐a$, $y\left(1\right)={\text{d}}_{FC}≐b$ and $y\left(2\right)={\text{d}}_{FA}+{\text{d}}_{FC}≐c$, $y\left(3\right)={\text{d}}_{F}≐d$, then its state-space representation reads:


(6) $$\begin{array}{l} \xi \left(k+1\right)=M\xi \left(k\right),\;k=0,1 \end{array}$$

with ξ(*l*) representing the vector [*y*(*l*), *y*(*l* + 1)]^T^, *l* = 0, 1, 2, and M denoting the square 2 × 2 matrix [0, 1;1, 1]. Here, notation *a, b, c, d* is used for the sake of simplicity. Currently, by repeatedly solving the system for *k* = 0, 1, one gets ξ(1)=Mξ(0), $\xi \left(2\right)=M\xi \left(1\right)=M^{2}\xi \left(0\right)$, with $M^{2}$ as [1, 1;1, 2]. Let $c_{\phi }={\left(1+\phi^{2}\right)}^{-1/2}$. As M is a symmetric real matrix with distinct eigenvalues {ϕ, 1 − ϕ} and orthogonal eigenvectors $v_{\phi }=c_{\phi }{\left[1,\phi \right]}^{\text{T}}$, $v_{\left(1-\phi \right)}=c_{\phi }{\left[\phi ,-1\right]}^{\text{T}}$, the solution in terms of *y*(2) and *y*(3) takes the explicit form


$$\begin{array}{l} \left[\begin{array}{c} y\left(2\right)\\ y\left(3\right) \end{array}\right]=\phi^{2}\beta v_{\phi }+{\left(1-\phi \right)}^{2}\alpha v_{\left(1-\phi \right)}, \end{array}$$

where α = 〈ξ(0), *v*_(1 − ϕ)_〉 = (ϕ*a* − *b*)*c*_ϕ_, β = 〈ξ(0), *v*_ϕ_〉 = (*a* + ϕ*b*)*c*_ϕ_ denote the projections of the initial condition ξ(0) along the directions of the two orthogonal eigenvectors. Meaningful properties are in order.

1. When α is equal to zero, i.e., when the initial vector ξ(0) has no components along the direction of the eigenvector *v*_(1−ϕ)_, the equality *b*/*a* = ϕ holds and the equalities *c*/*b* = ϕ, *d*/*c* = ϕ hold too.
2. In the general case in which *b*/*a* ≠ ϕ denote: *b*/*a* by α_0_; *c*/*b* by β_0_; *d*/*c* by γ_0_, and write *c*/*b* = β_0_ = (*a* + *b*)/*b* = 1 + 1/α_0_; *d*/*c* = γ_0_ = (*a* + 2*b*)/(*a* + *b*) = 1 + 1/β_0_; then get (recall that ϕ − 1 = 1/ϕ): ϕ − β_0_ = ϕ − 1 − 1/α_0_ = (α_0_ − ϕ)/(ϕα_0_); ϕ − γ_0_ = ϕ − 1 − 1/β_0_ = (β_0_ − ϕ)/(ϕβ_0_), and, as the two ratios *c*/*b* = β_0_ and *d*/*c* = γ_0_ are greater than 1 as follows: i) |ϕ − γ_0_| < |ϕ − β_0_|; ii) |ϕ − β_0_| < |ϕ − α_0_| when also *b*/*a* = α_0_ is greater than 1, hold.

Inequalities (i)–(ii) in 2. thus show that the last (third) ratio of the elements of the sequence (Equation 5) is closer to the golden ratio than the first and the second ones. Indeed, the last ratio associated with the generalized Fibonacci sequence becomes ever closer to the golden ratio once the length of such a sequence becomes longer and longer (for the same initial ratio). This is in accordance with the limit behavior


$$\begin{array}{l} \underset{k\to +\infty }{\lim}\frac{y\left(k+1\right)}{y\left(k\right)}=\underset{k\to +\infty }{\lim}\frac{\phi^{k+1}\beta c_{\phi }-{\left(1-\phi \right)}^{k}\alpha c_{\phi }}{\phi^{k}\beta c_{\phi }+{\left(1-\phi \right)}^{k}\phi \alpha c_{\phi }}\;=\;\phi , \end{array}$$

holding true for a generalized Fibonacci sequence of infinite length. The illustrative example in the Appendix will shed more light on the question. This means that by increasing the length of the Fibonacci sequence (thus reducing the number of sub-phase durations working as independent seeds), the ratios between consecutive elements come close to the golden ratio, with this being in line with the experimental evidence, in automatizable scenarios, regarding walking gait cycles of healthy subjects (see Verrelli et al., 2021a and references therein), as well as swimming strokes (Verrelli et al., 2021b,c, 2023) and tennis forehand executions (Verrelli et al., 2024): constraints coming from the temporal symmetrization process (iteratively leading to extended Fibonacci sequences), which are satisfied in all the subjects having carried out a sufficiently large number of cyclic movements (training in sports), make the last ratios approximate the golden ratio, with such a special number thus unfolding and becoming quite visible by experimental measurements.

#### 2.1.2 Time harmonization through exact self-similarity achievement (steps 3–4)

Come back to the generalized Fibonacci sequence: *a*, *b*, *c*, *d* of Theorem 1, with ${\text{d}}_{FA}=a$, ${\text{d}}_{FC}=b$ and ${\text{d}}_{FA}+{\text{d}}_{FC}=c$, ${\text{d}}_{F}=d$. If the last ratio *d*/*c* is imposed to equal ϕ, then, by direct computation,


(7) $$\begin{array}{l} \phi =\frac{d}{c}\;=\;\frac{c+b}{c}\;=\;1+\frac{b}{c} \end{array}$$

and then


$$\begin{array}{l} \phi -1=\frac{b}{c}\;=\;\frac{1}{\phi } \end{array}$$

leading to *c*/*b* = ϕ. By applying similar steps, *b*/*a* turns out to equal ϕ too. In other words, imposing the last ratio *d*/*c* to be equal to ϕ leads to a chain of equalities—involving ϕ—holding true for all the consecutive ratios *b*/*a*, *c*/*b*, *d*/*c*. In general, imposing the last ratio of a Fibonacci sequence of length *r* to be equal to ϕ leads to a chain of equalities—involving ϕ—holding true for all the *r* − 1 consecutive ratios and with one value—the first seed—determining the whole sequence. The resulting event F is a special event, among the ones generated by a self-referential loop and described by Theorem 1. When the second subprocess of this subsection, namely, the exact self-similarity enforcement, is additionally carried out after the first subprocess of the previous subsection, the brain will resort to the minimum amount of information (zero Shannon entropy) for a highly aesthetical movement temporal design. It will lead to building ever stronger automatic circuits of mental dependence on memory as soon as the length of the Fibonacci sequence increases. This is highlighted hereafter (taken, for the sake of exhaustiveness, from Verrelli et al., 2021a): the more different letters there are in a string, the more difficult it is to correctly predict which letter will be the next one in the same string. To this purpose, take the three differences *d*_1_ = *b*/*a* − *c*/*b*, *d*_2_ = *d*/*c* − *c*/*b*, *d*_3_ = *b*/*a* − *d*/*c* and let *M*_*R*_ be a sufficiently large positive odd integer. Let P be a finite partition of the compact set [−*M*_*R*_, *M*_*R*_], with disjoint blocks (or cells) $A_{j}$ of the form:


$$\begin{array}{l} A_{j}=\left[x_{j},x_{j+1}\right),\;j=1,2,\ldots ,M_{R}-1,\\ A_{j}=\left[x_{j},x_{j+1}\right],\;j=M_{R}, \end{array}$$

where *x*_*j*+1_ = *x*_*j*_ + 2, *j* = 1, 2, …, *M*_*R*_, and *x*_1_ = −*M*_*R*_. Let $P_{l}$ a finite refinement of P (*l* = 0, 1, …, *R*_*l*_, *R*_*l*_ is a sufficiently large natural number), with finer blocks $A_{k\left[j\right]}^{\left[l\right]}\subset A_{j}$ of the form:


$$\begin{array}{l} A_{k\left[j\right]}^{\left[l\right]}=\left[x_{k\left[j\right]}^{\left[l\right]},x_{k+1\left[j\right]}^{\left[l\right]}\right), \end{array}$$

where $x_{k+1\left[j\right]}^{\left[l\right]}=x_{k\left[j\right]}^{\left[l\right]}+1/2^{l}$, *k* = 1, …, 2^(*l*+1)^, and $x_{1\left[j\right]}^{\left[l\right]}=x_{j}$. For each *l* = 0, 1, …, *R*_*l*_, define the set of characters (or letters)


$$\begin{array}{l} \Sigma^{\left[l\right]}=\left\{x_{1}=x_{1\left[1\right]}^{\left[l\right]},\ldots ,x_{2^{\left(l+1\right)}+1\left[1\right]}^{\left[l\right]}=x_{2}=x_{1\left[2\right]}^{\left[l\right]},\ldots ,\ldots \right\}. \end{array}$$

Consider the string of characters: $\left(s_{1}^{\left[l\right]},s_{2}^{\left[l\right]},s_{3}^{\left[l\right]}\right)$, where $s_{1}^{\left[l\right]}$, $s_{2}^{\left[l\right]}$, $s_{3}^{\left[l\right]}$ belong to the above set Σ^[*l*]^ and $s_{m}^{\left[l\right]}$ (*m* = 1, 2, 3) equals the smallest element of $A_{k\left[j\right]}^{\left[l\right]}$ when the difference *d*_*m*_ belongs to the block $A_{k\left[j\right]}^{\left[l\right]}$. Let $p_{*r}^{\left[l\right]}$ be the number of characters belonging to the *r*-th character type in the three-elements-string $\left(s_{1}^{\left[l\right]},s_{2}^{\left[l\right]},s_{3}^{\left[l\right]}\right)$ divided by 3 (*r* = 1, …, *N*^[*l*]^, *N*^[*l*]^ ≤ 3). Finally, take the Shannon index for such a string $\left(s_{1}^{\left[l\right]},s_{2}^{\left[l\right]},s_{3}^{\left[l\right]}\right)$ as


$$\begin{array}{l} H_{s}^{\left[l\right]}=-\overset{N^{\left[l\right]}}{\underset{r=1}{\sum }}p_{*r}^{\left[l\right]}\text{ln}\left(p_{*r}^{\left[l\right]}\right), \end{array}$$

where $\overset{N^{\left[l\right]}}{\underset{r=1}{\sum }}p_{*r}^{\left[l\right]}=1$. The more unequal the abundances of types in the string are, the smaller the corresponding Shannon entropy is made, with $H_{s}^{\left[l\right]}$ satisfying


$$\begin{array}{l} H_{s}^{\left[l\right]}\in \left[0,\text{ln}\left(N^{\left[l\right]}\right)\right]. \end{array}$$

In particular, if all abundance is concentrated to one type, Shannon entropy is zero and there is no uncertainty in predicting the type of the next entity. The case in which the golden ratio ϕ is a fixed point for the consecutive ratios *b*/*a*, *c*/*b*, and *d*/*c* is thus characterized by the condition $\overset{R_{l}}{\underset{l=1}{\sum }}H_{s}^{\left[l\right]}=0$, for any *R*_*l*_ ∈ ℕ ∪ {0}.

### 2.2 Generation process (step *r*-*r* + 1)

The same logical scheme (Definition-Theorem) of the previous subsection can be further extended to the step *r*-*r* + 1. It suffices to look at:

- The role of the aggregate phase FA∪FB whose duration appears as the second element of Fib_3_ of Equation 3;
- Its partition FA, FB and at the corresponding symmetrization step in which the duration of the longest subphase of FA∪FB is imposed to equal the first element of Fib_3_ of Equation 3;
- The Fibonacci sequence extension in which the duration of the shortest subphase of FA∪FB is used as a new first element of Fib_4_ of Equation 5 extending Fib_3_ of Equation 3.

In other words, at each step, the symmetric sub-partitioning leading to the elementary pattern $P_{A}$ is iteratively applied. Thus, it comes to the following Definition 2 and Theorem 2 (its proof is straightforward at this stage, including the non-decreasing nature of the resulting Fibonacci sequence) generalizing Definition 1 and Theorem 1:

**Definition 2**: The *r* − 3*-times-Fibonacci-left-extendable* event F, consisting of *r* phases F1,…,Fr=F whose durations compose a (non-decreasing) Fibonacci sequence Fib_*r*_ of length *r*:


(8) $$\begin{array}{l} {\text{Fib}}_{r}:{\text{d}}_{1},{\text{d}}_{2},\ldots ,{\text{d}}_{r} \end{array}$$

is *r* − 2*-times-Fibonacci-left-extendable* if F2 can be partitioned into F2a, F2b with durations d_2*a*_ < d_2*b*_ satisfying the symmetrization step


(9) $$\begin{array}{l} {\text{d}}_{2b}={\text{d}}_{1}. \end{array}$$

**Theorem 2**: The generalized (non-decreasing) Fibonacci sequence of length *r* + 1 (extending Fib_*r*_ of Equation 8 to the left)


(10) $$\begin{array}{l} {\text{Fib}}_{r+1}:{\text{d}}_{2a},{\text{d}}_{1}={\text{d}}_{2b},{\text{d}}_{2},\ldots ,{\text{d}}_{r} \end{array}$$

is associated with the *r* − 2*-times-Fibonacci-left-extendable* event F of Definition 2.

For what the second subprocess enforcing self-similarity is concerned, when the length of the Fibonacci sequence increases (along the symmetrization direction) the self-similarity constraint might involve an ever larger number of ratios. Indeed the level of aesthetic perception of the gesture increases with the number of characters in the string related to the Shannon index as the number of independently determined durations of the subphases constituting the gesture converges to one. Accordingly, the *power of self-similarity* can be defined. It depends on the number of ratios involved in the self-similarity constraint as well as, necessarily, on the length of the involved Fibonacci sequence. This is again in line with the experimental evidence regarding walking gait cycles of healthy subjects—as well as swimming strokes and tennis forehand executions—and resembles the different levels of self-similarity there introduced: self-similarity of power 1, which involves 3 ratios for a generalized 4-length Fibonacci sequence, is called simple self-similarity, while self-similarities of power 2 or 3, which involve 4 or 5 ratios for generalized 5- and 6-length Fibonacci sequences, are referred to as strong self-similarity and enhanced self-similarity (see Verrelli et al., 2021b), respectively. Higher powers for self-similarity—coming from higher lengths of Fibonacci sequences—constitute more advanced versions of self-similarity as they correspond to ever better sewn sub-phases with ever higher aesthetical features. Indeed, when the entire gesture is captured by a generalized Fibonacci sequence (namely, all the sub-phases of the gesture are mapped into a generalized Fibonacci sequence of their durations) and all the ratios of consecutive elements of the sequence are equal to the golden ratio, the perceiver catches the presence of a single, well-defined, highly aesthetical, uniform pattern within the entire gesture.

*Remark 1*: It is worth mentioning that if the general step described by Definition 2 is applied from the beginning to an event F which the sequence


$$\begin{array}{l} d,{\text{d}}_{F} \end{array}$$

corresponds to, with *d* yet to be determined, the procedure turns out to automatically generate (Equation 3). In this light, the described process is self-generating from the very first step.

*Remark 2*: If, at each step, the partitioning regarding the sub-phase, whose duration appears as the second element of the Fibonacci sequence to be extended, also applies to the symmetric phase, then the extension to the left can be viewed as a *strong extension to the left*. In the example regarding the human walking gait cycle, this corresponds, for example, to a partition of both the swing phases in which both of their longest sub-phases last as the double support.

*Remark 3*: The two distinct sub-processes of the procedure described in this paper, as two increasing levels of automatization, use the addition operation to generate the Fibonacci sequence and the product operation to enforce self-similarity. The first subprocess symmetrizes the phase durations, while the second one enforces self-similarity. It is clear that iteratively sub-partitioning each phase (starting from the event itself) while enforcing the self-similarity constraint at each step coincides with the sequential application of the two sub-processes.

## 3 Application to the walking gait

The results of the previous section have a direct application to the walking scenario. Consider the walking gait cycle of a healthy subject at a comfortable speed of Verrelli et al. (2021a) and apply the general procedure so far described. Thus, start from F representing the gait cycle, FA representing the double support, FB, FC representing the swing phases. Therefore, the gait cycle is as follows:

1. Once-Fibonacci-extendable if the swing phases have the same duration (generally the double support phase duration is smaller than the swing phase duration);
2. Twice-Fibonacci-extendable if the swing phase can be partitioned into two subphases with the longest one lasting like the double support;
3. 3-times-Fibonacci-extendable if the double support phase can be partitioned into two phases with the longest one lasting like the shortest subphase of the swing phase.

A simple illustrative numerical example is here given for a 3-times-Fibonacci-extendable gait cycle whose duration is 1. This is in line with the conventional gait analysis that expresses the walking phases with respect to the gait cycle (100%, i.e., Equation 1), defined from a foot strike to the subsequent strike of the same foot on the ground. Indeed, all the walking phases are reported as percentages of the gait cycle. The procedure of the previous section involving the temporal symmetrization generates the 6-length sequence


(11) $$\begin{array}{l} 0.10,0.14,0.24,0.38,0.62,1.00 \end{array}$$

starting from an original 3-length sequence 0.38, 0.62, 1.00. Equation 11 is not perfectly self-similar, though the last ratio is rather close to ϕ. Indeed, it approximates the self-similarly partitioned sequence reading:


(12) $$\begin{array}{l} 0.09018,0.14591,0.23608,0.38198,0.61804,1.00000. \end{array}$$

Currently, the above conditions 1.-3. for such a sequence are reviewed as follows.

- Condition 1. corresponds to a symmetrical gait cycle in which the percentage durations of both the swing phases are equal to 38.198%, the first starting at **11.804** = 23.608/2% of the gait cycle, the second starting at **61.806** = 23.608+38.198%.
- Condition 2. corresponds to a partition of the second swing phase (percentage-wise lasting 38.1984) with boundary event at 14.591% of such a swing phase (and thus at **76.395**% of the gait cycle).
- Condition 3. corresponds to a partition of the double support phase with boundary event at 7.2955 = 14.591/2% of each portion of the double support phase (the first occurring at **7.2955**%, the second occurring at **57.2975** = 11.804+38.198+7.2955%).

**Figure 1:**
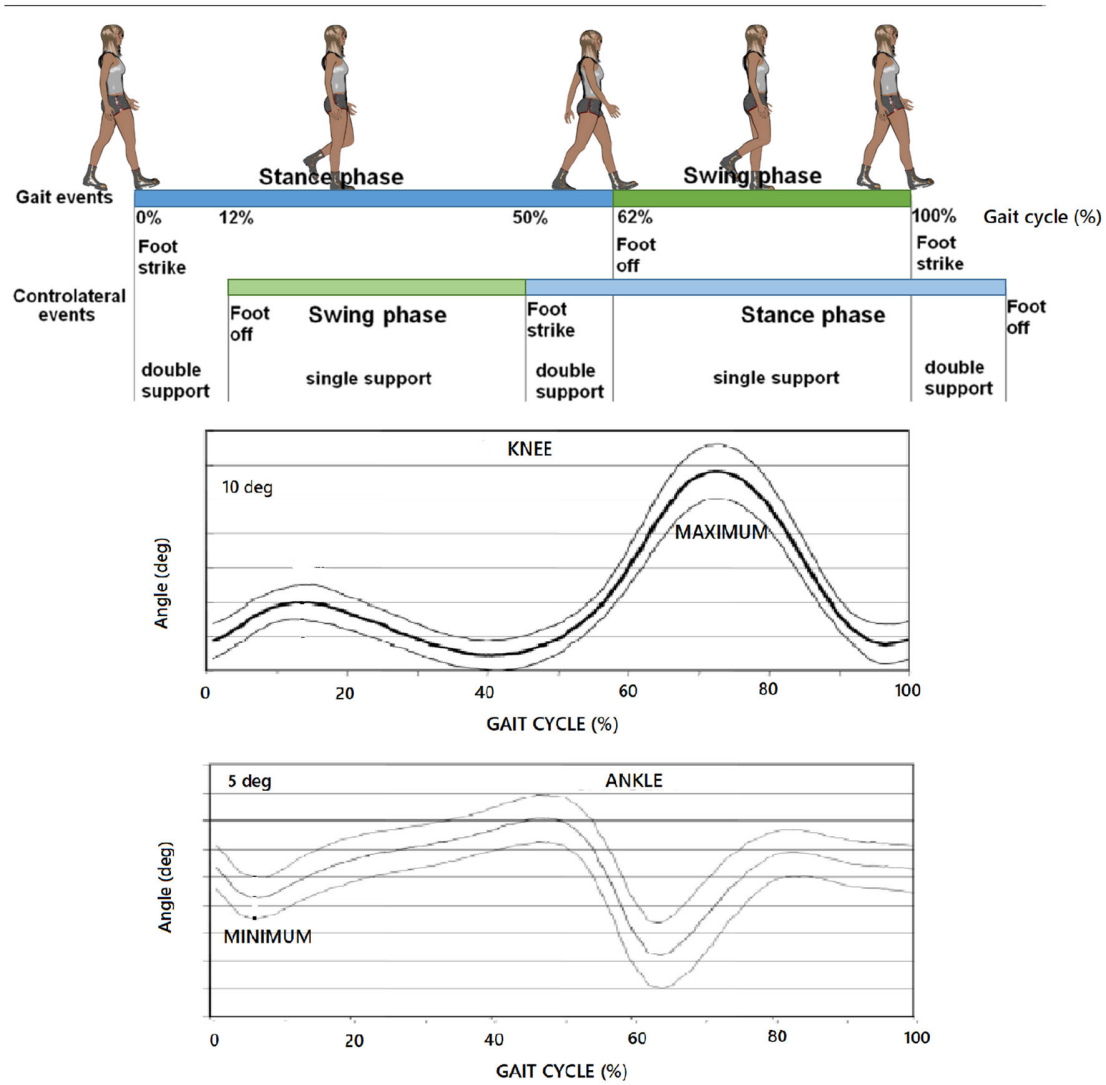
Typical partition (at a comfortable speed) of: gait cycle; typical knee profile; typical ankle profile. Partitions coming from Condition 2 and Condition 3 here refer to (i) the knee flexion peak and (ii) the instant of the minimum position of the foot relative to the tibia with 90 degrees-angle being plotted at 0-degrees, respectively.

Such partitions of the last two items exhibit a physical meaning (Novacheck, 1998) as soon as they identify (i) the knee flexion peak and (ii) the instant of the minimum position of the foot relative to the tibia with 90 degrees-angle being plotted at 0-degrees, as the boundary events previously introduced, respectively. While the latter reduces to the matter of the conjecture in Verrelli et al. (2021a), the former is brand new and surprisingly complies with Figure 1, which reports the typical percentages that are associated with a gait cycle at speeds around the comfortable one.

**Figure 2:**
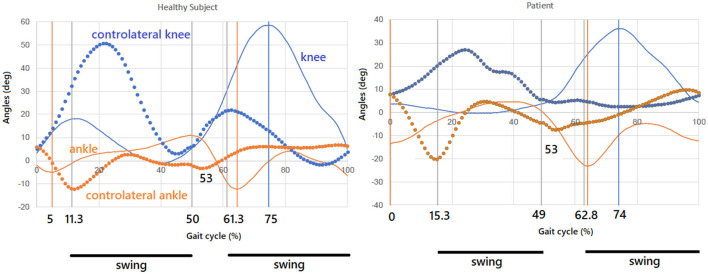
Healthy subject and pathological subject: Partition of the gait cycle with knee–ankle profiles. Mechanization and self-similarity level of the healthy subject is here largely higher than the pathological subject and closer to the sequence in Equation 12.

Nevertheless, Figure 2 reports the percentages that are associated, in our experiments, with a healthy subject and a pathological one (a case of muscular dystrophy) walking at a comfortable speed. As can be seen, though both the levels of coordinative mechanization and harmonicity are not totally accomplished, the mechanization and self-similarity level of the healthy subject is largely higher than the pathological subject and it turns out to be closer to the sequence in Equation 12 [compare the percentages reported there to the bold ones appearing in the previous text].

Differently from top-rank tennis players of the next section, who get ever higher levels of coordinative mechanization and self-similarization through repetitive training aimed at continuously improving performance, general healthy subjects settle for—but do not definitely improve—their satisfactory mechanization and self-similarization levels, as a perfectly mechanized and self-similar gait cycle (at comfortable speed) is not generally connected to a competitive advantage. Nevertheless, lower levels associated with pathological gaits are in line with the results of Iosa et al. (2007) and, more in general, of Iosa et al. (2016).

## 4 Application to the tennis forehand

The main discoveries from Verrelli et al. (2024) are recalled here for the sake of clarity. The contribution of this section consists of showing how the constraints that were introduced there are here found to coincide with the conditions 1.-2. (once-Fibonacci extendability, twice-Fibonacci extendability) so far discussed and this actually sheds more light on paper (Verrelli et al., 2024).

The forehand stroke is characterized by the following five time instants that belong to the modern forehand stroke and are not conditioned by personalism and high-level initial muscle co-activation (see Figures in Verrelli et al., 2024): TI1: swing start point of maximum loading (point of maximum racket height); TI2: shoulder rotation, beginning shoulder line rotation; TI3: impact; TI4: R180, 180 deg rotation of the racket after impact (the point where the racket cap faces the camera); TI5: final, time instant when the kinetic energy of the blow is exhausted.

As reported in Verrelli et al. (2024), they define the following four phases (the total duration PS of the stroke is the duration of the phase from TI1 to TI5): Phase *i*: from TI*i* to TI*i* + 1, with duration P*i* (fraction of the forehand stroke duration PS), *i* = 1, …, 4. Apply the procedure presented in this paper starting from the 3-length generalized Fibonacci sequence


$$\begin{array}{l} \text{P}3+\text{P}4,\text{ P}2+\text{P}1,\;1, \end{array}$$

so that

- The 4-length generalized Fibonacci sequence (steps 3–4 with P1 being longer than P2)
(13) $$\begin{array}{l} \text{P}2,\text{ P}3+\text{P}4,\text{ P}2+\text{P}1,\;1 \end{array}$$
  is obtained under the constraint (once-Fibonacci-extendability):
(14) $$\begin{array}{l} C_{1}:\text{ P}3+\text{P}4-\text{P}1=0; \end{array}$$
- The 5-length generalized Fibonacci sequence (steps 4–5 with P4 being longer than P3)
(15) $$\begin{array}{l} \text{P}3,\text{ P}2,\text{ P}3+\text{P}4,\text{ P}2+\text{P}1,\;1 \end{array}$$
  is obtained under the additional constraint (twice-Fibonacci-extendability):
(16) $$\begin{array}{l} C_{2}:\text{ P}4-\text{P}2=0. \end{array}$$

Indeed, the first constraint is nothing but condition 1., while the second constraint is nothing but condition 2. They quantify (namely, the absolute values of P3+P4-P1 and P4-P2) the level of coordinative mechanization as previously meant (0 represents the maximum level of coordinative mechanization). On the other hand, the self-similarity level of the two above sequences is quantified by the two Φ-bonacci indices in Verrelli et al. (2024):


$$\begin{array}{l} I_{f,4}=100[{\left(\text{P}1+\text{P}2-0.61804\right)}^{2}+{\left(\text{P}1-0.38198\right)}^{2}\\ +{\left(\text{P}2-0.23608\right)}^{2}{]}^{1/2}\\ I_{f,5}=100[{\left(\text{P}1+\text{P}2-0.61804\right)}^{2}+{\left(\text{P}1-0.38198\right)}^{2}\\ +{\left(\text{P}2-0.23608\right)}^{2}+\\ {\left(\text{P}3-0.14591\right)}^{2}{]}^{1/2} \end{array}$$

comparing consecutive ratios of sub-phase durations in the Fibonacci sequences (Equations 13, 15) to the golden ratio ϕ. The smaller such indices are, the stronger the level of self-similarity results for such sequences. They rely on the following two propositions (adapted from Verrelli et al., 2024) providing the harmonic percentages of the Fibonacci sequences (Equations 13, 15).

**Proposition 1:** If P_1_/P_2_ = ϕ, then sequence (Equation 13) exhibits an internal self-similar structure, with percentage duration of Phase 1 and Phases 3-4 ≊38.198%, percentage duration of Phase 2 ≊23.608%.

**Proposition 2:** If P_2_/P_3_ = ϕ, then sequence (Equation 15) exhibits an internal enhanced self-similar structure, with percentage duration of Phase 1 ≊38.198%, percentage duration of Phase 2 and Phase 4 ≊23.608%, percentage duration of Phase 3 ≊14.591%.

Currently, it is original to analyze, in the new light provided by this paper, the measurements in Verrelli et al. (2024) in terms of the level of coordinative mechanization achieved by Amateurs players and Advanced players.^1^ Those data are also complemented here by (analogously obtained) timing data concerning two top-rank players, namely, TP1 (male) and TP2 (female), performing successful forehand strokes in the most challenging scenario of a (public—ATP Master 1000 and WTA 1000) training rally (strokes not requiring lateral/frontal movements of the entire body are selected).^2^ They are reported in Table 1 while the corresponding phase percentages are in Table 2. They lead to the values for the constraints (Equations 14, 16) and for the Φ-bonacci indices highlighted in Table 3. It is straightforward to recognize, on the basis of such values, a high level of coordinative mechanization for both TP1 and TP2. The reader might also appreciate the stability of the stroke timing in the challenging scenario of a training rally. Meanwhile, self-similarity in TP2 is preserved even under a stroke with a larger duration, as the timing of the following stroke [s]—not reported in the Tables—0.000, 0.375, 0.600, 0.725, 0.946 (and percentages 39.6, 23.8, 13.2, 23.4) reveals. Such new data from top-rank tennis players turn out to be crucial in identifying high-quality automatized and harmonic strokes. Furthermore, stroke 2 of TP2 (whose relative rank is higher than TP1) demonstrates that a highly automatized and self-similarized stroke exists in real practice and, actually, in the challenging scenario of a training rally.

**Table 1:** Time instants TI1-TI5 for top-rank players TP1 (male) and TP2 (female).

	**TI1 (s)**	**TI2 (s)**	**TI3 (s)**	**TI4 (s)**	**TI5 (s)**
TP1 (stroke 1)	0.00	0.333	0.583	0.704	0.950
TP1 (stroke 2)	0.00	0.354	0.552	0.658	0.850
TP1 (stroke 3)	0.00	0.308	0.500	0.612	0.812
TP2 (stroke 1)	0.00	0.308	0.508	0.620	0.825
TP2 (stroke 2)	0.00	0.308	0.500	0.616	0.808
TP2 (stroke 3)	0.00	0.312	0.512	0.629	0.829

**Table 2:** Percentage durations of Phases 1–4 for top-rank players TP1 (male) and TP2 (female).

	**P1 (%)**	**P2 (%)**	**P3 (%)**	**P4 (%)**
TP1 (stroke 1)	35.05	26.32	12.74	25.89
TP1 (stroke 2)	41.65	23.29	12.47	22.59
TP1 (stroke 3)	37.93	23.65	13.79	24.63
TP2 (stroke 1)	37.33	24.24	13.58	24.85
TP2 (stroke 2)	38.12	23.76	14.36	23.76
TP2 (stroke 3)	37.64	24.13	14.11	24.13

**Table 3:** Top-rank tennis players TP1 and TP2: values for constraints (Equations 14, 16) and for Φ-bonacci indices representing the levels of coordinative mechanization and self-similarization (three strokes per player).

**TP1**	**Stroke 1**	**Stroke 2**	**Stroke 3**	**RMS**
$C_{1}$ (%)	3.579	−6.588	0.493	2.376
$C_{2}$ (%)	−0.421	−0.706	0.985	0.406
$I_{f,4}$ (%)	4.173	4.673	0.353	1.984
$I_{f,5}$ (%)	4.567	5.132	0.872	2.190
**TP2**	**Stroke 1**	**Stroke 2**	**Stroke 3**	**RMS**
$C_{1}$ (%)	1.091	0.000	0.603	0.394
$C_{2}$ (%)	0.606	0.000	0.000	0.192
$I_{f,4}$ (%)	1.096	0.190	0.765	0.427
$I_{f,5}$ (%)	1.494	0.302	0.902	0.560

A high level of coordinative mechanization occurs for both TP1 and TP2. Stroke 2 of TP2 (whose relative rank is higher than TP1) here demonstrates that a highly automatized and self-similarized stroke exists in real practice, in the challenging scenario of a training rally.

**Figure 3:**
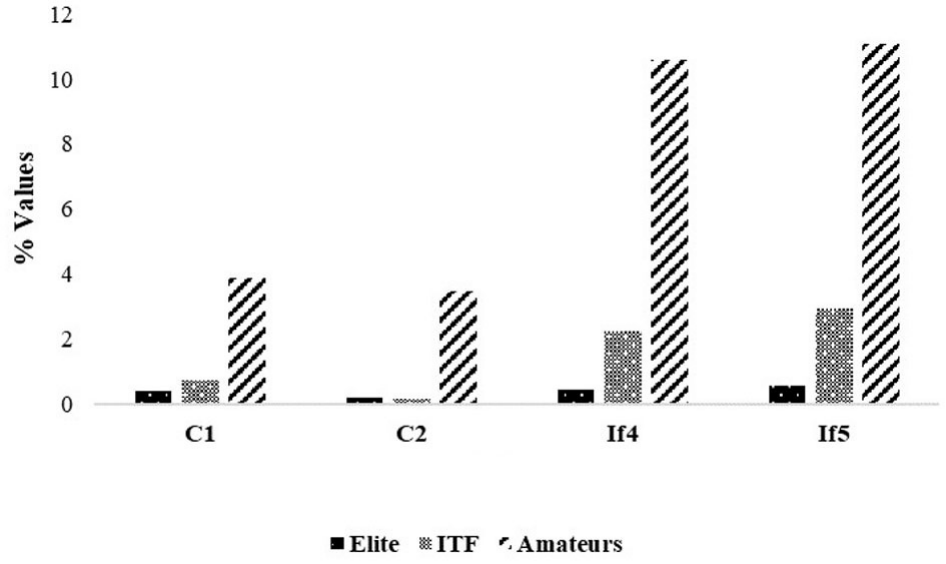
Levels of coordinative mechanization and self-similarization within the three groups: amateurs, advanced, top-rank players (RMS stands for root mean square). The levels of coordinative mechanization and self-similarization increase with the quality of players (statistically significant differences were found between the groups in all the variables—*p* < 0.001—by using the Kruskal–Wallis Test).

This is further confirmed by Figure 3, which definitely shows how the levels of coordinative mechanization and self-similarization increase—namely, the related indices decrease—with the quality of players. In particular, the level of coordinative mechanization of advanced players is closer to top-ranking players than amateurs. However, differently from top-rank players, advanced and amateurs players' data do not concern the challenging training rally scenario but a simple basket drill (closed-skill situation). Furthermore, the results of the correlation analysis related to all the players' strokes in terms of Spearman correlations:

- $I_{f,5}$-$C_{1}$: 0.828 (*p* < 0.001)

- $I_{f,5}$-$C_{2}$: 0.737 (*p* < 0.001)

- $I_{f,5}$-$I_{f,4}$: 0.957 (*p* < 0.001)

- $C_{1}$-$C_{2}$: 0.528 (*p* < 0.001)

- $C_{1}$-$I_{f,4}$: 0.869 (*p* < 0.001)

- $C_{2}$-$I_{f,4}$: 0.688 (*p* < 0.001)

show how strong Spearman correlations were found among all the investigated parameters except between constraints $C_{1}$ and $C_{2}$, for which a moderate correlation was found. This seems to move along the direction of viewing a tennis player first improving, along the repetitions of strokes, the level of coordinative mechanization (with no priority given to one of the two constraints) and just then increasing the level of self-similarity (with the highest priority given to lower powers of self-similarity over higher ones).

## 5 Extension to the tennis serve

**Figure 4:**
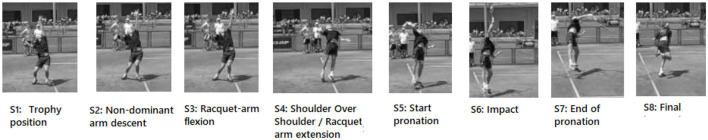
Eight instants characterizing the tennis serve. S1: Trophy position; S2: Non-dominant arm descent; S3: Racquet-arm flexion; S4: Shoulder Over Shoulder / Racquet arm extension; S5: Start pronation; S6: Impact; S7: End of pronation; S8: Final.

This section is dedicated to originally showing how the automatic generation process described in this paper is also at the root of hidden self-similar patterns that appear in the tennis serve (under no external disturbances). The conceptual disjunction between the symmetrization-based Fibonacci sequence generation and the time harmonization achieved through exact self-similarity enforcement is then illustrated by the serve performed by the top-rank tennis player TP1 (see the previous section). First, we define, within the serve swing movement, the following eight instants, common in all players (free from personalism): S1: Trophy position: instant of maximum loading; S2: Non-dominant arm descent: beginning of the downward movement of the non-dominant arm and the tilting of the shoulder line; S3: Racquet-arm flexion: start of the racquet acceleration phase, characterized by flexion of the dominant arm; S4: Shoulder Over Shoulder/Racquet arm extension: shoulder line overturn and start of arm extension; S5: Start pronation: start of forearm pronation movement; S6: Impact: instant the racket impacts the ball; S7: End of pronation: end of forearm pronation movement; S8: Final: instant in which the kinetic energy of the stroke is exhausted. They are reported and illustrated in Figure 4. They define the following seven phases (the total duration PS of the serve swing is the duration of the entire serve movement from S1 to S8): Phase *i*: from S*i* to S*i* + 1, with percentage duration P*i*, *i* = 1, …, 7.

Apply the procedure presented in this paper starting from the three-length generalized Fibonacci sequence


(17) $$\begin{array}{c} \text{P}3+\text{P}4+\text{P}6,\text{ P}1+\text{P}2+\text{P}5+\text{P}7,\;1, \end{array}$$

so that the four-length generalized Fibonacci sequence (steps 3–4 with P7 being longer than P1 + P2 + P5)


(18) $$\begin{array}{c} \text{P}1+\text{P}2+\text{P}5,\text{ P}3+\text{P}4+\text{P}6,\text{ P}1+\text{P}2+\text{P}5+\text{P}7,\;1 \end{array}$$

is obtained under the constraint (once-Fibonacci-extendability of sequence (17)):


(19) $$\begin{array}{c} C_{1}:\;\left(\text{P}3+\text{P}4+\text{P}6\right)-\text{P}7=0; \end{array}$$

the five-length generalized Fibonacci sequence (steps 4–5 with P3 being longer than P4+P6)


(20) $$\begin{array}{c} \text{P}4+\text{P}6,\text{ P}1+\text{P}2+\text{P}5,\text{ P}3+\text{P}4+\text{P}6,\text{ P}1+\text{P}2+\text{P}5+\text{P}7,\;1 \end{array}$$

is obtained under the additional constraint [twice-Fibonacci-extendability of sequence (Equation 18)]:


(21) $$\begin{array}{c} C_{2}:\;\left(\text{P}1+\text{P}2+\text{P}5\right)-\text{P}3=0; \end{array}$$

the six-length generalized Fibonacci sequence (steps 5–6 with P1 being longer than P2+P5)


(22) $$\begin{array}{c} \text{P}2+\text{P}5,\text{ P}4+\text{P}6,\text{ P}1+\text{P}2+\text{P}5,\text{ P}3+\text{P}4+\text{P}6,\\ \text{ P}1+\text{P}2+\text{P}5+\text{P}7,\;1 \end{array}$$

is obtained under the additional constraint [three-times-Fibonacci-extendability of sequence (Equation 20)]:


(23) $$\begin{array}{c} C_{3}:\;\left(\text{P}4+\text{P}6\right)-\text{P}1=0; \end{array}$$

the seven-length generalized Fibonacci sequence (steps 6–7 with P6 being longer than P4)


(24) $$\begin{array}{c} \text{P}4,\text{ P}2+\text{P}5,\text{ P}4+\text{P}6,\text{ P}1+\text{P}2+\text{P}5,\text{ P}3+\text{P}4+\text{P}6,\\ \text{ P}1+\text{P}2+\text{P}5+\text{P}7,\;1 \end{array}$$

is obtained under the additional constraint [four-times-Fibonacci-extendability of sequence (Equation 22)]:


(25) $$\begin{array}{c} C_{4}:\;\left(\text{P}2+\text{P}5\right)-\text{P}6=0; \end{array}$$

the eight-length generalized Fibonacci sequence (steps 7–8 with P5 being longer than P2)


(26) $$\begin{array}{c} \text{P}2,\text{ P}4,\text{ P}2+\text{P}5,\text{ P}4+\text{P}6,\text{ P}1+\text{P}2+\text{P}5,\text{ P}3+\text{P}4+\text{P}6,\\ \text{ P}1+\text{P}2+\text{P}5+\text{P}7,\;1 \end{array}$$

is obtained under the additional constraint [five-times-Fibonacci-extendability of sequence (Equation 24)]:


(27) $$\begin{array}{c} C_{5}:\text{ P}5-\text{P}4=0. \end{array}$$

Currently, the time instants S2–S8 corresponding to a (stable) serve swing performed by the top-rank tennis player TP1 (same setup as the previous section) are reported in Table 4 (S1 = 0). First, by inspecting the five constraints, it turns out that they take the values:


$$\begin{array}{c} C_{1}:\text{ P}3+\text{P}4+\text{P}6=\text{P}7,↔37.5302≊38.8620\\ C_{2}:\text{ P}1+\text{P}2+\text{P}5=\text{P}3,↔23.6077≊23.6077\\ C_{3}:\text{ P}4+\text{P}6=\text{P}1,↔13.9295≊12.1065\\ C_{4}:\text{ P}2+\text{P}5=\text{P}6,↔11.5012≊9.0799\\ C_{5}:\text{ P}5=\text{P}4,↔7.2639≊4.8426. \end{array}$$

**Table 4:** Time instants S2–S8 top-rank player TP1 (male) and related phase percentages (S1 = 0).

**S2 (s)**	**S3 (s)**	**S4 (s)**	**S5 (s)**	**S6 (s)**	**S7 (s)**	**S8 (s)**
0.100	0.135	0.330	0.370	0.430	0.505	0.826
**P1 (%)**	**P2 (%)**	**P3 (%)**	**P4 (%)**	**P5 (%)**	**P6 (%)**	**P7 (%)**
12.1065	4.2373	23.6077	4.8426	7.2639	9.0799	38.6620

The first three ones quite precisely hold true (the second one perfectly matches), whereas the last two ones are less precisely satisfied, though corresponding to relatively small time discrepancies (PS = 0.826 s). Second, the generalized Fibonacci sequences are computed in accordance with Equations 18, 20, 22, 24, 26. In particular, the four-length generalized Fibonacci sequence (under $C_{1}$) reads


(28) $$\begin{array}{c} 23.6077,\;37.5302,\;61.1379,\;≊100; \end{array}$$

the five-length generalized Fibonacci sequence (under $C_{2}$) reads


(29) $$\begin{array}{c} 13.9295,\;23.6077,\;37.5302,\;61.1379,\;≊100; \end{array}$$

the six-length generalized Fibonacci sequence (under $C_{3}$) reads


(30) $$\begin{array}{c} 11.5012,\;13.9295,\;23.6077,\;37.5302,\;61.1379,\;≊100; \end{array}$$

the seven-length generalized Fibonacci sequence (under $C_{4}$) turns out to be


(31) $$\begin{array}{c} 4.8426,\;11.5012,\;13.9295,\;23.6077,\;37.5302,\;61.1379,\\ \;≊100; \end{array}$$

the eight-length generalized Fibonacci sequence (under $C_{5}$) turns out to be


(32) $$\begin{array}{c} 4.2373,\;4.8426,\;11.5012,\;13.9295,\;23.6077,\;37.5302,\\ \;61.1379,\;≊100. \end{array}$$

Meaningfully, they well-approximate the self-similarly partitioned sequence reading:


(33) $$\begin{array}{c} 3.445,5.573,9.018,14.591,23.608,38.198,61.804,100, \end{array}$$

which self-similarity of power 5 corresponds to. On the other hand, the previous derivations lead to a rather interesting enrichment of the analysis, which is illustrated in Table 5. Such a table reports the harmonic durations and percentages that would represent the most mechanized and harmonic version of the same serve swing performed by TP1 and described in Table 4. They are explicitly computed by means of Equations 17–27, 33.

**Table 5:** Time instants S2–S8 and related phase percentages (S1 = 0) for the fully automatized and harmonic version of the same serve swing by TP1 in Table 4.

**S2 (s)**	**S3 (s)**	**S4 (s)**	**S5 (s)**	**S6 (s)**	**S7 (s)**	**S8 (s)**
0.12052	0.14898	0.34398	0.39001	0.43604	0.5105	0.826
**P1 (%)**	**P2 (%)**	**P3 (%)**	**P4 (%)**	**P5 (%)**	**P6 (%)**	**P7(%)**
14.591	3.445	23.608	5.5573	5.5573	9.018	38.198

Such durations and percentages, explicitly computed by means of Equations 17–27, 33, would represent the most automatized and harmonic version of the same serve swing performed by TP1 of Table 4.

Apparently, the discrepancies between durations (s) in Table 4 and computed values (s) in Table 5 have just a (maximum) 0.02-magnitude, while the video analysis procedure allows the operator to manually identify instants affected by 0.004 errors (s) [camera shooting mode 1,920 × 1,080 px at a sampling rate of 240 fps; inaccuracy of the internal clock oscillator of the camera less than 0.1μs]. It is worth emphasizing that the partition of Table 5 is highly aesthetical as it corresponds to a self-similarity of power 5 for a generalized Fibonacci sequence of length 8 (namely, Equation 33).

## 6 Discussion

This paper addresses the analysis of complex repetitive human movements (or parts of them) that allow for a converging iterative sub-partitioning procedure to be gradually and progressively achieved through learning (Singer, 2002), with coordinative mechanization and self-similarization playing distinct crucial roles. In such a light, this paper addresses the analysis of complex repetitive human movements (or parts of them) that allow for an iterative sub-partitioning procedure subject to a bilateral (such as in walking) or unilateral (such as in tennis) process of temporal symmetrization. This commonly happens when motion is mainly characterized by (i) alternation of flexor and extensor muscle activities in the sagittal plane or (ii) alternation of abduction and adduction phases in the lateral plane or (iii) activities of internal and external rotator muscles in the transverse plane. Currently, the recent results discussed in Section 1 have suggested that humans resort to the same Fibonacci sequence-based criterion to automatize repetitive movements associated with both the lower limbs (walking) and the upper limbs (tennis). This fact can be explained by recognizing that our brain has to optimize motor control by reducing the biomechanical degrees of freedom for each limb, with this being similar in upper and lower limbs (3 for shoulder and hip, 1 for elbow and knee, and 2 for ankle and wrist). Nevertheless, mechanization (involving all the sub-phases of the gesture) and self-similarity generation, as increasing levels of automatization of the entire gesture, tend to progressively reduce Shannon entropy: the ratios between the durations of consecutive sub-phases in the associated Fibonacci sequence tend to be restricted to the same value so that the brain can resort to the minimum amount of information for the movement temporal design (just one sub-phase duration) in a highly aesthetical fashion. This, in turn, is reasonably connected to fluidity maximization and rigidity minimization (Feletti et al., 2023), as one sub-phase duration of a complex movement is able to aesthetically generate an entire sequence of sub-phase durations of the same movement as it happens in perfectly sewn sub-movements. Obviously, Shannon entropy and rigidity minimizations are mapped through a non-injective and surjective mapping of spatial position/speed profiles into phase durations, which necessarily requires the analysis of this paper to complement a spatial angle-based analysis of the gesture. Indeed, the findings of this paper may have practical implications across various fields. As aforementioned, from a neurophysiological perspective, the subdivision of a movement into phases that maintain the same proportion with their subphases appears to be an optimization strategy for simplifying motor control and addressing the motor equivalence problem associated with the coordinated control of numerous possible degrees of freedom (Goldberger, 1996). Dominici et al. (2011) found that the development of independent walking starts from two locomotor primitives already present in the stepping reflex furtherly developed into four primitives that may correspond to the stride phases: two double support phases, single support, and swing. The first independent steps of toddlers seem to act as a trigger for developing a self-similar structure of these gait phases based on the golden ratio (Bartolo et al., 2022). This trend, although slower and asymmetric, was also observed in children with cerebral palsy and could be used to assess walking deficits (Bartolo et al., 2024). The presence of this fractal structure may be relevant not only in the development of walking but also in the recovery of locomotor function during rehabilitation. Some examples of this approach already exist. The distance between gait phase ratios and the golden ratio can be measured to assess locomotor impairment and even used to treat patients. Several studies have demonstrated the efficacy of providing rhythmic acoustic stimuli to patients with Parkinson's disease to support the timing of their walking. Belluscio et al. (2021) provided external auditory cues whose timing was based on the golden ratio, finding that these cues partially compensated for the defective internal rhythm of the basal ganglia in Parkinson's disease. Additionally, Tez and Kuscu (2017) constructed a humanoid robot that walked according to the golden proportion, observing that this approach resulted in a smoother gait. Furthermore, considering walking gait rehabilitation and sports training as degenerate (re-) learning processes, it is crucial to employ repetitive strategies that facilitate the recovery of specific movement patterns. Dzeladini et al. (2014) utilized a computerized neuromuscular skeletal model capable of auto-adapting its parameters to simulate human walking and noted that the model naturally converged toward a configuration in which the golden ratio equals the ratio between successive gait phases. This concept may also influence sports training by encouraging athletes to adapt their motor patterns, over repetitions, toward self-similar structures, particularly in closed-skill sports such as swimming or in open-skill sports with specific gestures that can be considered closed skills, such as the tennis forehand and serve, of this paper. With this respect, although movement learning is a continuous process, three stages can be identified to characterize events during the early, middle, and late stages in the development of a single motor activity. They are in order:

(1) The cognitive stage occurs at the beginning of learning. Attempts are made to understand the character of the motor activity to be learned. In this stage, much thinking is needed, understanding the intent and purposes of certain motor actions and devising techniques to achieve the goals.

(2) The associative stage is the intermediate stage, in which the learner understands what needs to be done.

(3) The autonomous phase is considered the final one in the process toward skill acquisition. The behavior is automatic and there is minimal conscious control over movement (Salehi et al., 2021). Indeed, the final stage is combined with automaticity and presents self-control and direction as in the mechanization process of this paper.

An additional related issue regards the connection between movement dynamics and underlying neural processes. In particular, further studies should investigate the Fibonacci sequence- based patterns presented in this paper from the neuroscience point of view, with specific attention to the brain neuroplasticity—in terms of neural reconfigurations, attentional processes, and cognitive efforts—at the root of the temporal Fibonacci sequence generation and harmonization. In this respect, the dynamic reconfiguration of the brain networks occurring during learning has been found to predict the relative amount of learning in the future sessions (Bassett et al., 2011), with the attention given to the new task and the related cognitive effort being progressively reduced when the mechanism becomes more automatic. Indeed, current findings show that the development of walking is a locomotor learning process that is based on the brain network adaptation being triggered by the experience of the first independent steps. After them, the gait–phase ratios in toddlers rapidly seem to converge to the golden value (Bartolo et al., 2022). In children with cerebral palsy, instead, an overall harmonic walking pattern is developed, but more slowly and asymmetrically in the two legs (Bartolo et al., 2024). It is worth noticing, as well, that brain reconfiguration during the learning processes has been also investigated, *via* electroencephalography, in cognitive tasks regarding language (Mariani et al., 2023) or thinking (Jia and Zeng, 2021) and in the Stroop task (Barzon et al., 2024). Studying the electrical activity of the brain using electroencephalography (Thompson et al., 2008) is also crucial for understanding the processes behind learning and skillful execution of sporting gestures. Electroencephalography is in fact an excellent non-invasive technique to investigate psychomotor efficiency (Hatfield et al., 2020) and study the neural mechanisms of sports performance during training (Cheng and Hung, 2020a,b). In spite of possible limitations (Thompson et al., 2008; Tharawadeepimuk and Yodchanan, 2021; Fang et al., 2022)—brain data might be contaminated by artifacts of non-brain origin, for example, due to muscle activity, especially during exercise—new state-of-the-art amplifier and headset systems allow for good accuracy in recording during exercise and sports (di Fronso et al., 2019), electroencephalography could be applied, in future developments, to enhance understanding of the processes behind learning automatized sports movements while investigating what happens in sports gestures that respect harmonic temporal structures and how athletes' mental states are related to automation and harmonicity, and consequently performance (Cheng et al., 2024).

On the other hand, the fluidity of movements, which appears to be related to the harmonic self-similarity of this paper, actually refers to how smoothly and seamlessly actions are performed, with no abrupt transitions or jerky motions. Motions blend into the next ones naturally, whereas high control over muscular tension and relaxation is required to obtain steady and graceful movement. Indeed, synchronization achieves well-coordinated movements, which appear effortless and connected, as well as graceful and elegant, whereas movements that match a rhythm or are timed correctly relative to other actions avoid being choppy, with tension being minimized even in complex or demanding motions. This can be achieved only when motor behavior is advantageously influenced by attention, perception, memory, and memory-based decision-making, which suitably balances muscle activation and co-activation within the gesture while getting large benefits from the prolonged practice. Indeed, it is the muscle co-activation that affects movement mechanics in optimizing movement and providing stability during various physical activities (Latash, 2018). It refers to the simultaneous activation of agonist and antagonist muscles useful for motor control, to avoid over-extension during rapid and explosive movements. It thus influences movement efficiency. This phenomenon has been quantified using several indices, usually based on direct recording of muscle activation of both muscles within an agonist–antagonist pair. The co-activation index (CoI), for instance, compares the activation of the antagonist muscle (or muscle group) to the activation of the agonist muscle (or muscle group) or analyzes combined agonist and antagonist activation. In particular, research indicates that muscle co-activation plays a vital role in the coordination of movements to maintain postural control, especially in the presence of high cognitive load or environmental challenges. High levels of muscle co-activation have been shown to occur in healthy subjects with fatigue (Wang et al., 2015), whereas, in sports, high levels of CoI can lead to increased joint stiffness and stability, Latash (2018), Salem et al. (2021), and Akl et al. (2021) and may reduce the risk of injury (Hirokawa et al., 1991; Lehman, 2006; Knudson and Blackwell, 1997). However, excessive CoI can lead to increased metabolic costs and reduced movement efficiency, indicating a delicate balance that must be maintained for optimal performance (Mian et al., 2006). The relationship between co-activation and movement optimization is further underscored by studies that explore the effects of training and rehabilitation on co-activation patterns, suggesting that targeted interventions can enhance motor control and functional outcomes (Palazzo et al., 2022). Nevertheless, some studies (Wang et al., 2019; Pizzamiglio et al., 2017) indicate that expert athletes could have a different muscle activation pattern with less antagonist muscle activation, implying that antagonistic muscle coupling might be altered by specialized activity. As a result, especially during fast movements, athletes may have lower muscle co-activation than non-athletes (Bazzucchi et al., 2008). In tennis (Rota et al., 2012; Tai et al., 2022) and other racquet sports, for instance, CoI of elbow muscles could be used as an indicator of coordination between agonist and antagonist muscle activity during three phases of the shot (Akl et al., 2021). Greater levels of CoI in the arm muscles were found during fast compared to slow movements, increasing in the preparation phase (neglected by our previous analysis of tennis forehand and serve), low during execution to allow for greater acceleration, and increasing at the end of the movement to provide dynamic braking and inhibit elbow extension (Bazzucchi et al., 2006; Rouard and Clarys, 1995). Decreasing muscle co-activation during the execution phase enables the generation of greater speed and increased performance (Bazzucchi et al., 2008; Akl et al., 2021). It is clear, however, that even though balancing muscle activation and co-activation to maximize fluidity seems to be conceptually related to Shannon entropy minimization (with duration ratios as characters) and in line with the results of the present study, such results are to be interpreted with caution, owing to its specific limits, such as the small size of the samples analyzed in the experimental sets. Involving the achievement of experimental evidence that confirms the theoretical connection between the effects of the mechanization and self-similarization process and the fluidity improvements will be the goal of future study.

## 7 Conclusion

A solution has been provided to the open problem of providing a theoretical understanding of the automatic generation process at the root of hidden self-similar patterns appearing in cyclic human movements (walking, running, swimming, and tennis-playing) within movement-automatization-allowable scenarios under no external disturbances or additional constraints. An original conceptual disjunction between the symmetrization-based Fibonacci sequence generation (coordinative mechanization) and the time harmonization through self-similarity enforcement has been presented. They represent two increasing levels of automatization, with the latter enhancing, refining, and perfecting the former. This explains how cyclic human movements can progressively achieve: (i) sequence length-based increasing levels of coordinative mechanization, with self-similarity as a just asymptotic feature; (ii) minimization of the Shannon entropy with ever higher aesthetic (highly technical) characteristics at increasing powers of self-similarity. Theoretical results have been illustrated in detail *via* data concerning walking and tennis playing [though results are to be interpreted with caution, owing to its specific limits, such as the small size of the samples analyzed in the experimental sets]. They provide support to explain how people suitably determine the timing of complex repetitive movements that are characterized by a high number of degrees of freedom while shedding light on how motor behavior is influenced by cognitive processes such as attention, perception, memory, and decision-making. In our view, even though relationships between Shannon entropy minimization (coming from the mechanization & self-similarization process) and muscle activation and co-activation optimal balance (to maximize fluidity) are to be covered by future experimental works, the temporal concepts outlined in this paper might relevantly be applied to both sports training and rehabilitation while encouraging athletes and patients to adapt or recover their motor patterns, over repetitions, toward self-similar structures.

## Data Availability

The raw data supporting the conclusions of this article will be made available by the authors, without undue reservation.

## Footnotes

^1^For the sake of exhaustiveness, the study involved 11 subjects (9 males and 2 females), divided based on their technical quality into two groups: advanced (*n*=8 age: 21.3± 4) and amateurs (*n*=3 age: 32.7 ± 6.8). The players in the advanced group all had an international ATP (Association of Tennis Professionals) or ITF (International Tennis Federation) ranking on the day of sampling. Those in the Amateurs' group had no ranking, neither national nor international. All the subjects performed 10 longline forehands (two sets of five repetitions) on easy balls fed by the coach from the basket. A WOLFANG GA200 camera set at 720px 240fps and GOPRO hero 11 in HD at 240fps were used for video shooting. The cameras were placed approximately 6 m behind and in addition to the player's impact position (back and side perspectives), respectively, and at a height of 1.10 m above the ground. In this regard, it is worth mentioning that line $C_{2}$ of Figure 6 in Verrelli et al. (2024) contains some (here amended) typos (as well as Phase 5 in place of Phase 4 before Proposition 1) while constraint values are not percentages in Figures 6, 7 of Verrelli et al. (2024).

^2^Additional data—not reported here for the sake of brevity—confirm that a distortion of the coordinative mechanization and self-similarization processes occurs as soon as external disturbances or additional constraints act on the player.

## Appendix

### Illustrative example for golden ratio generation

First, consider a 3-length (non-decreasing) generalized Fibonacci sequence of the form (the reader can think of the percentage durations of swing, stance, and gait cycle in order)

$$\begin{array}{c} x_{1},\;x_{2},\;x_{3}=100. \end{array}$$

The only constraints are here given by *x*_1_ > 0, *x*_2_ ≥ *x*_1_ and *x*_1_ + *x*_2_ = *x*_3_. The previous sequence can be thus rewritten as

$$\begin{array}{c} x_{1},\;x_{2},\;x_{1}+x_{2}=100 \end{array}$$

so *x*_1_ must belong to the set **(0, 50]** while *x*_2_ must belong to the set **[50, 100)**. Currently, assume that you can extend the previous sequence to the left, in accordance with the generation step of Section 2.2, and get

$$\begin{array}{c} x_{0},\;x_{1},\;x_{2},\;x_{3}=100. \end{array}$$

The additional couple of constraints would be given by *x*_1_ ≥ *x*_0_ and *x*_0_ + *x*_1_ = *x*_2_, allowing us to rewrite the previous sequence as

$$\begin{array}{c} x_{0},\;x_{1},x_{0}+x_{1},\;x_{0}+2x_{1}=100, \end{array}$$

with this leading to i) $x_{0}\leq 100/3=33.\bar{3}$, $x_{1}\geq 100/3=33.\bar{3}$, $x_{2}=100-x_{1}\leq 66.\bar{6}$, ii) *x*_0_ > 0, *x*_1_ < 50 and *x*_2_ = 100−*x*_1_ > 50. The procedure would have thus narrowed the range of the sequence elements *x*_1_, *x*_2_ from (0, 50] and [50, 100) to $\text{[}33.\bar{3},50)$ and $\text{(}50,66.\bar{6}]$ (while $x_{0}\in \text{(}0,33.\bar{3}]$). It is interesting to also evaluate what would happen if the previous sequence could be further extended to the left. It would read

$$\begin{array}{c} x_{+},\;x_{0},\;x_{1},\;x_{2},\;x_{3}=100 \end{array}$$

while inheriting the additional constraints *x*_+_ > 0, *x*_0_ ≥ *x*_+_, and *x*_+_ + *x*_0_ = *x*_1_. Analogously, rewriting the previous sequence as

$$\begin{array}{c} x_{+},\;x_{0},\;x_{+}+x_{0},\;x_{+}+2x_{0}=x_{0}+x_{1},\;2x_{+}+3x_{0}\\ =x_{0}+2x_{1}=x_{1}+x_{2}=100 \end{array}$$

would lead to (i) *x*_+_ ≤ 100/5 = 20, *x*_0_ ≥ 100/5 = 20, *x*_1_ = (100−*x*_0_)/2 ≤ 40, *x*_2_ = 100−*x*_1_ ≥ 60, (ii) *x*_+_ > 0, $x_{0}=\left(100-2x_{+}\right)/3<100/3=33.\bar{3}$, $x_{1}=\left(100-x_{0}\right)/2>100/3=33.\bar{3}$, $x_{2}=100-x_{1}<66.\bar{6}$. As such, *x*_2_ would be reduced to belong to the set $\text{[}60,66.\bar{6})$, *x*_1_ to $\text{(}33.\bar{3},40]$, *x*_0_ to $\text{[}20,33.\bar{3})$ (while *x*_+_ ∈ (0, 20]). The same reasoning would happen in the case in which the previous sequence can be further extended to the left, namely, into

$$\begin{array}{c} x_{-},\;x_{+},\;x_{0},\;x_{1},\;x_{2},\;x_{3}=100 \end{array}$$

under the additional constraints *x*_−_ > 0, *x*_+_ ≥ *x*_−_ and *x*_−_ + *x*_+_ = *x*_0_. Analogous computations can be used to show that *x*_2_ would be reduced to belong to the set **(60, 62.5]**, *x*_1_ to **[37.5, 40]**, *x*_0_ to **(20, 25]**, *x*_+_ to **[12.5, 20)** (while *x*_−_ ∈ **(0, 12.5]**). It is clear that, with the increase in the number of sub-partitioning steps, the reduction in the size of the sets, to which the elements of the increasing-length sequence belong to, performs a sort of convergence toward the self-similarity scenario.
